# Positive Coping and Resilience as Mediators Between Negative Symptoms and Disability Among Patients With Schizophrenia

**DOI:** 10.3389/fpsyt.2019.00641

**Published:** 2019-09-10

**Authors:** Haotian Chen, Jianfeng Xu, Yue Mao, Lili Sun, Yujing Sun, Yuqiu Zhou

**Affiliations:** ^1^Nursing Department, The Second Affiliated Hospital Zhejiang University School of Medicine (SAHZU), Hangzhou, China; ^2^School of Nursing, Harbin Medical University, Daqing, China; ^3^Department of Senior Citizens Welfare, Beijing College of Social Administration, Beijing, China

**Keywords:** resilience, coping skill, personal resources, psychosis, schizophrenia, disability

## Abstract

**Objective:** This study proposes a schizophrenia disability model to describe the associations between negative symptoms and disability to test the possible mediating roles of positive coping and resilience and to compare the relative weights of the indirect effects of these two mediators in an integrated whole.

**Methods:** A total of 407 hospitalized Han Chinese patients diagnosed with stable schizophrenia or schizoaffective disorder were included. Patients were evaluated using the following scales: the Simplified Coping Style Questionnaire (SCQ) for positive coping, the Connor-Davidson Resilience Scale (CD-RISC) for resilience, the Positive and Negative Syndrome Scale (PANSS) for negative symptoms, and the World Health Organization Disability Assessment Schedule, Version II (WHO-DAS II) for the severity of disability. The schizophrenia disability distal mediation model was constructed using the structural modeling (SEM) approach. Bootstrapping procedures and the PRODCLIN program were used to examine the mediating roles of positive coping and resilience.

**Results:** The schizophrenia disability model was well-fitted to the observed data. Positive coping and resilience together with negative symptoms explained 66% of the variance in disability. Positive coping and resilience partly mediated the negative symptoms–disability relationship. The bootstrapped unstandardized indirect effect was 0.319, and the direct effect was 0.224. Positive coping also has a significant positive effect on resilience. In addition, the ratio of the specific indirect effect of positive coping to the total indirect effect (48%) is higher than that of resilience (30%).

**Conclusion:** Positive coping and resilience are two key causal mediators of the negative symptoms–disability relationship. Positive coping and resilience are important personal resources for patients with schizophrenia. We found that the indirect effect of positive coping was relatively more important than that of resilience. This result suggests that personalized treatments aimed at resilience and positive coping can effectively buffer the impact of negative symptoms for patients with schizophrenia and promote rehabilitation.

## Introduction

Schizophrenia is among the most disabling disorders worldwide. Patients with schizophrenia have a wide range of deficits in their everyday functioning (1). Only approximately 40% of patients may experience a considerable improvement in functioning from the onset of psychosis, and more than 80% of patients with schizophrenia experience permanent disability (2). Negative symptoms have been identified as the main drivers of disability in patients with schizophrenia and are significantly better predictors than all other symptom domains, such as psychotic symptoms (3, 4). Approximately two-thirds of patients who achieve symptomatic remission continue to experience persistent problems with functioning (5). Recovery in schizophrenia refers to not only remaining free of psychopathology but also regaining social and vocational functions and returning to the community. Recently, some studies have suggested that patients with a similar severity of psychopathology may have different functional outcomes because of differences in personal resources (6, 7). Therefore, identifying the role of personal resources in the disability process of patients with schizophrenia may be an important step in developing effective targeted interventions that may offer new ways to reduce the impact of negative symptoms on disability and promote rehabilitation.

In the last few years, resilience has been considered a crucial personal resource and a therapeutic factor in psychiatry. Resilience refers to the ability to regain or maintain mental health and to positively adapt to adversity and challenges (8, 9). Resilience must be considered a multidimensional and dynamic construct that helps individuals redesign the relationship between their family and social and external support systems rather than a unitary construct (10). Resilience can positively influence real-life functioning and is considered a protective factor that guarantees a good outcome for patients with psychosis (11). Highly resilient individuals demonstrate adaptive psychological and physiological responses and maintain psychobiological allostasis when experiencing adverse events, which are extremely common in schizophrenia (12). Two long-term follow-up studies have clarified the close relationship between resilience and positive outcomes in patients with schizophrenia. A 15-year long-term follow-up study conducted by Harrow and Jobe (13) found that protective factors, including greater resilience, a favorable personality, and attitudinal approaches, contribute to better outcomes in patients with schizophrenia. In addition, resilience and a good personality allow recovered patients to maintain a state of recovery after 20 years, even without medication (14). Zizolfi et al. (15) found that resilience factors may predict the severity of symptoms and the extent of psychosocial functioning and are considered an intervening variable between psychopathology and global functioning. Galderisi et al. (6) also found support for the hypothesis that resilience partially mediates the relationship between avolition and real-life functioning. In Mihali’s et al. (16) resilience theory, the effects of resilience, which is conferred by environmental, genetic, and social factors, can preclude, reverse, or slow the progression of schizophrenia. However, it remains unclear how resilience protects patients from disability and whether resilience works by buffering damage from negative symptoms.

Coping is a complex interaction between the individual and the environment that can be distinguished into emotion-focused coping, problem-focused coping, and avoidance-focused coping. Generally, problem-focused coping is associated with better outcomes and is therefore described as positive coping (17). Patients with schizophrenia who have serious negative symptoms might be characterized as more dependent on passive, emotion-focused coping, such as neglecting the problem, than on problem-focused coping when they face adversities. Patients with more severe schizophrenia symptoms often have poor outcomes partly because of their reduced use of positive coping strategies (18). Boschi et al. (19) also documented that adaptive coping is the best means to promote better functional outcomes for patients with schizophrenia. In addition, the severity of psychiatric symptoms is inversely correlated with positive coping, which in turn is correlated with functional outcomes (7).

Although coping and resilience are similar and the terms are used interchangeably, there is growing consensus that resilience and coping are distinct but related constructs. Gooding et al. (10) qualitatively interviewed 23 schizophrenia patients who had expressed suicidal thoughts and behaviors and found that an active response can combat negative stressors and is an effective psychological mechanism to promote resilience. A study of 200 postdoctoral scholars from a large research institution revealed that positive coping serves as an important mediator of the relationship between positive emotions and resilience and that an increase in positive coping can build resilience (20). However, the issues of whether positive coping and resilience are distinct constructs, whether positive coping is linked to resilience in patients with schizophrenia patients, and how these characteristics work together in disability have not been thoroughly explored.

**Figure 1:**
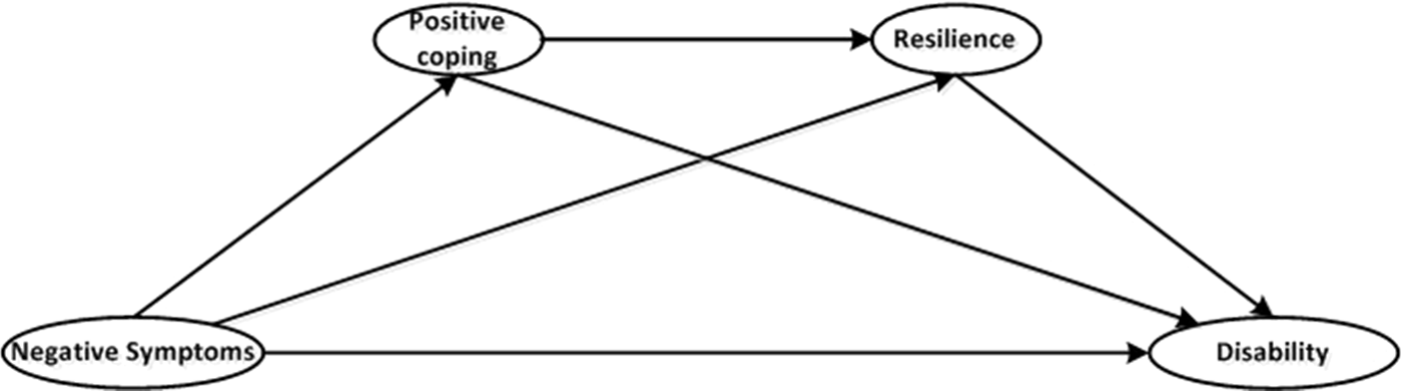
Proposed schizophrenia disability distal mediation model.

Previous studies have paid attention to only a single mediator and have neglected the existence of interactions among mediators. This gap hinders the ability to fully understand the mechanisms of these personal resources in buffering the influence of negative symptoms on disability and to promote more effective and efficient intervention measures. Given the theoretical and empirical evidence, we propose a schizophrenia disability model (**Figure 1**) to describe the associations among negative symptoms, positive coping, resilience, and disability to test the possible mediating role of positive coping and resilience and to compare the relative weights of the indirect effects of these two mediators as an integral whole. We hypothesize that negative symptoms will have direct effects on disability, as previous studies have demonstrated, and will have indirect effects on disability that are mediated by positive coping and resilience. We further hypothesize that positive coping also has a direct influence on resilience.

## Materials and Methods

### Procedures and Participants

Participants were recruited from the Third People’s Hospital of Daqing, which is a neurosis faculty hospital in the Heilongjiang province of China. Data were collected from March 2014 to March 2015. The participants were assessed to determine their coping style, resilience, and disability using interviewer-assisted and self-report methods. Psychiatric symptoms were assessed by two well-trained and experienced research psychiatrists who were not involved in the patients’ treatment and were blinded to all coping styles, resilience, and disability scores for the duration of the study. Patients with schizophrenia or schizoaffective disorder, as diagnosed by experienced research psychiatrists using the Structured Clinical Interview for the DSM-IV, were recruited after being referred by their clinicians. All patients were aged 18 years or above and signed a general written informed consent form. All patients were not taking antidepressants or mood stabilizers and had been in stable remission for at least 3 months. Participants were excluded if they were diagnosed with diseases known to affect neurocognition, such as Alzheimer’s disease, if they had a history of substance abuse or dependence, such as alcohol drinking, in the 3 months preceding enrollment, or if they were undergoing treatments, such as modified electroconvulsive therapy (MECT), that made them unable to complete the assessments. All participants received a complete description of our study and provided written informed consent. Our study received approval from the medical ethics committee of Harbin Medical University (Daqing) and strictly followed the principles of the Declaration of Helsinki.

### Measures

#### Positive Coping

The most widely used measures of coping style are the 66-item Ways of Coping Questionnaire (WCQ) (21) and the 30-item Coping Style Questionnaire (CSQ) (22). However, Chinese researchers have determined that these tools are not appropriate for Chinese populations because of the inconsistent factor analysis results. In our study, positive coping was assessed with the Simplified Coping Style Questionnaire (SCQ) developed by Xie (23). The SCQ is based on the WCQ and the characteristics of the Chinese population. It is a 20-item self-report questionnaire with two categories: positive coping styles (1–12 items, e.g., confiding in others) and negative coping styles (13–20 items, e.g., escaping troubles by taking a break). For the purpose of our study, the negative coping subscale was omitted, and only the positive coping subscale (SCQ-P) was used. The participants rated each item from 0 (never) to 3 (often) based on the frequency with which they used a given strategy when addressing a stressful situation or problem. A previous study reported that the positive coping scale had good reliability (24).

#### Resilience

Resilience was measured using the Connor-Davidson Resilience Scale (CD-RISC), which is a 25-item, 5-point Likert-type scale ranging from 0 (not true at all) to 4 (true all the time) (25). The participants rated each item based on how they felt over the previous month. The total score ranges from 0 to 100, with higher scores representing greater resilience. Given the possible differences in the factor structure of resilience in people with American and Chinese cultural backgrounds, we used the 3-factor structure (26), which is more meaningful to Chinese people than the 5-factor structure suggested by Connor and Davidson. The 3-factor structure comprises tenacity, strength, and optimism, all of which have adequate internal reliability (0.88, 0.80, and 0.60, respectively). The Chinese version of the CD-RISC also has good internal consistency (26).

#### Negative Symptoms

The Positive and Negative Syndrome Scale (PANSS) is currently the most widely used symptom measure in schizophrenia research settings. The PANSS instrument includes 30 items that were originally organized into three mutually exclusive subscales: positive symptoms (7 items), negative symptoms (7 items), and general psychopathology (16 items). Each symptom is rated on a 7-point scale from 1 (absent) to 7 (extreme). In the current study, we used the negative symptom scale of the PANSS to assess negative symptoms. We used analysis of variance (ANOVA) to examine interrater reliability, and psychiatrists with an intraclass correlation coefficient lower than 0.90 were excluded prior to the study. The remaining psychiatrists were trained, and reliability was retested at least once a month to maintain high interrater reliability.

#### Disability

We used the World Health Organization Disability Assessment Schedule, Version II (WHO-DAS II) to assess disability in patients with schizophrenia (27). The WHO-DAS II is a multidimensional instrument that provides a more accurate assessment of functional outcomes and disabilities in patients with schizophrenia compared with traditional instruments used to assess functioning (28). Because it is frequently difficult for hospitalized patients with schizophrenia to maintain employment, we selected the WHO-DAS II’s alternate 32-item scoring, which omits four items in the life activities domain related to work situations. The 32-item WHO-DAS II consists of six domains: cognition (six items), mobility (five items), self-care (four items), getting along (five items), life activities (four items), and participation (eight items). The patients were asked to rate each item from 1 (none) to 5 (extreme/cannot do) based on how much difficulty they had in the last month. The summary scores vary from 0 to 160 and are calculated by adding the ratings for each item, as described in the WHO-DAS manual (29). Higher scores reflect greater disability. It has previously been demonstrated that the Chinese version of the WHO-DAS II has good validity and reliability (30).

### Statistical Analyses

Raw data for normality, outliers, and missing values were assessed prior to the analyses. We also used the raw data to calculate a variance–covariance matrix to avoid inaccurate standard errors, as described by Cudeck (31). Correlation analysis was performed using the Pearson correlation test.

According to the “two-step approach” recommended by Anderson and Gerbing (32), we evaluated the measurement model and the structural model sequentially. Confirmatory factor analysis (CFA) was performed first to test whether the observed variables accurately reflected each of the underlying latent variables and to test the convergent validity and discriminant validity of the measurement model.

Following the measurement model, we used structural equation modeling (SEM), which can model multiple latent variables simultaneously while considering the reliability of their indicators, to examine the antecedents and consequences of the four proposed latent variables (one exogenous: negative symptoms; and three endogenous: positive coping, resilience, and disability). The model fit was evaluated with five indicators: the normed chi-square (χ*^2^* */df*), the comparative fit index (CFI), the incremental fit index (IFI), the Tucker-Lewis Index (TFI), and the root mean-squared error of approximation (RMSEA). A good-fitting model requires the following standard indices: χ*^2^* */df* between 1 and 3 and IFI, TLI, and CFI greater than 0.90. In addition, the 90% confidence interval of the RMSEA should be under 0.08 (33–35).

Our schizophrenia disability distal mediation model consisted of three specific indirect effects. Positive coping and resilience served as a single mediator between negative symptoms and disability, respectively, in the first and second specific indirect effects. In the third specific indirect effect, both positive coping and resilience mediated the relationship between negative symptoms and disability. The total indirect effect, direct effect, and total effect between negative symptoms and disability were tested using bootstrapping procedures. Two thousand samples were requested for bootstrapping, and the bias-corrected confidence interval (CI) was set to 95%. Because all of the major SEM software packages can only estimate the total indirect effects and not specific indirect effects (36), the significance of each specific indirect effect in our distal mediation model was also tested using the PRODCLIN program (37). All statistical analyses were performed with SPSS version 22.0 and Amos version 24.0.

## Results

### Demographic and Clinical Data

A total of 407 unrelated hospitalized patients with schizophrenia or schizoaffective disorder were recruited. All patients were Han Chinese with an average age of 39.3 (SD 10.6) years; 53.6% were male, and 31% were married. Their average total number of hospitalizations was 4.3 (SD 4.3), and their average course of schizophrenia was 11.7 (SD 9.9) years (for details, see **Table 1**). All of the included participants were receiving antipsychotic treatments (14% with a typical antipsychotic only, 78% with an atypical antipsychotic only, 8% mixed). The average PANSS total score was 61.8 (SD 14.4), the average CD-RISC score was 54.4 (SD 22.5), the average SCQ-P total score was 18.7 (SD 9.1), the average SAPS total score was 26.4 (SD 19), and the average WHO-DAS II total score was 66.9 (SD 19.6), as shown in **Table 1**.

**Table 1:** Sociodemographic characteristics and the means and standard deviations (SDs) of clinical characteristics (*n* = 407).

Characteristics	Mean	SD
**Demographics**		
Age (years)	39.3	10.6
Male (%)	53.6	
Married (%)	31.0	
Duration of illness (years)	11.7	9.9
Number of hospitalizations	4.3	4.3
**Symptoms (PANSS)**	61.8	14.4
PANSS Positive Symptoms	12.9	5.4
PANSS Negative Symptoms	17.8	6.2
PANSS General Symptoms	31.1	10.7
**Resilience (CD-RISC)**	54.4	22.5
Tenacity	24.0	11.9
Strength	19.6	8.7
Optimism	5.9	3.4
**Positive coping style (SCQ-P)**	18.7	9.1
**Disability (WHO-DAS II)**	66.9	19.6
Cognition	10.9	4.3
Mobility	7.9	2.9
Self-care	6.8	3.0
Getting along	8.4	3.3
Life activities	7.2	3.1
Participation	18.8	6.7

SCQ-P, the positive coping subscale of the Simplified Coping Style Questionnaire; CD-RISC, the Connor-Davidson Resilience Scale; PANSS, The Positive and Negative Syndrome Scale; WHO-DAS II, the World Health Organization Disability Assessment Schedule, Version II.

### Preliminary Analyses

No outliers or missing values were recorded in our raw data. All of the items had a normal distribution when tested for skewness and kurtosis. The results of the Pearson correlation test show significant correlations among all of the variables. Negative symptoms were negatively related to positive coping and resilience and were positively related to disability. Positive coping was negatively related to disability and was positively related to resilience. Resilience was negatively related to disability (for details, see **Table 2**). According to the sample size calculation tables provided by Fritz and Mackinnon (38) and our results from the structural model, the smallest path coefficients from exogenous variance to the mediator (r = -0.35) and the smallest path coefficients from the mediator to endogenous variance (r = -0.34) are the H level (the path coefficient of the H level is 0.26). A total of 148 participants for a bias-corrected bootstrap or 161 participants for the PRODCLIN program constitute a sufficient sample to achieve an empirical power of 0.8. Therefore, our sample size of 407 participants exceeded the required size.

**Table 2:** Correlation analysis of study variables.

Variables	1	2	3	4
**1. Negative symptoms**	1			
**2. Positive coping styles**	-0.45***	1		
**3. Resilience**	-0.51**	0.62***	1	
**4. Disability**	0.56***	-0.68***	-0.64***	1

***p < 0.001; **p < 0.01.

### Measurement Model

A good measurement of the latent variables is a necessary precondition for a causal relations analysis of the latent variables. Therefore, we first applied CFA to test the confidence of the relationship between the observed variables and the underlying latent variables. Items with factor loadings lower than 0.60, which indicates a lack of reliability (39), were discarded, as recommended by Hooper et al. (40). Three items for disability, two for positive coping, and two for resilience were excluded from further analyses (the factor loadings were between 0.43 and 0.52).

The construct convergent reliability and discriminant validity were measured. The composite reliability (CR) was between 0.84 and 0.98, and the average variance extracted (AVE) of the latent variables was between 0.55 and 0.63. All four constructs in our study had good convergent validity (39, 41). The AVE analysis showed that the AVE value of each latent variable was much larger than the square of Pearson’s correlation coefficient for each pair of latent variables, which indicates that our study had good discriminant validity (41) (**Table 3**).

**Table 3:** Results of the average variance extracted (AVE) analysis and the composite reliability (CR) of the latent variables.

Variables	1	2	3	4	CR
					
**1. Negative symptoms**	0.55				0.98
**2. Positive coping styles**	0.20	0.59			0.93
**3. Resilience**	0.26	0.39	0.63		0.84
**4. Disability**	0.31	0.46	0.41	0.63	0.91
					

The shaded portion of the table is the AVE value, and the square of Pearson’s correlation coefficient is shown below the AVE value.

For disability, 29 items within six subdimensions remained after the unreliable items were discarded. We used the first-order and second-order CFA. To determine the fit with the data, we computed the target coefficient, which is the ratio of the chi-square of the first-order CFA to the chi-square of the second-order CFA, according to Marsh (42). The closer the target coefficient is to 1, the closer the second-order CFA is to the first-order CFA. The target coefficient of disability of 0.98 indicates that the second-order CFA explained 98% of the variation in the first-order CFA of disability. Therefore, the fitness index of the second-order CFA of disability was good. The measurement model for resilience consisted of three first-order factors that were identified. Although this model could not be distinguished in a statistical sense, all of the standardized second-order factor loadings were between 0.77 and 0.85, above the loading of 0.70 recommended by Hair et al. (39). Therefore, we used the second-order CFA of resilience and disability instead of the first-order CFA to make the model more precise.

### Structural Model

**Figure 2:**
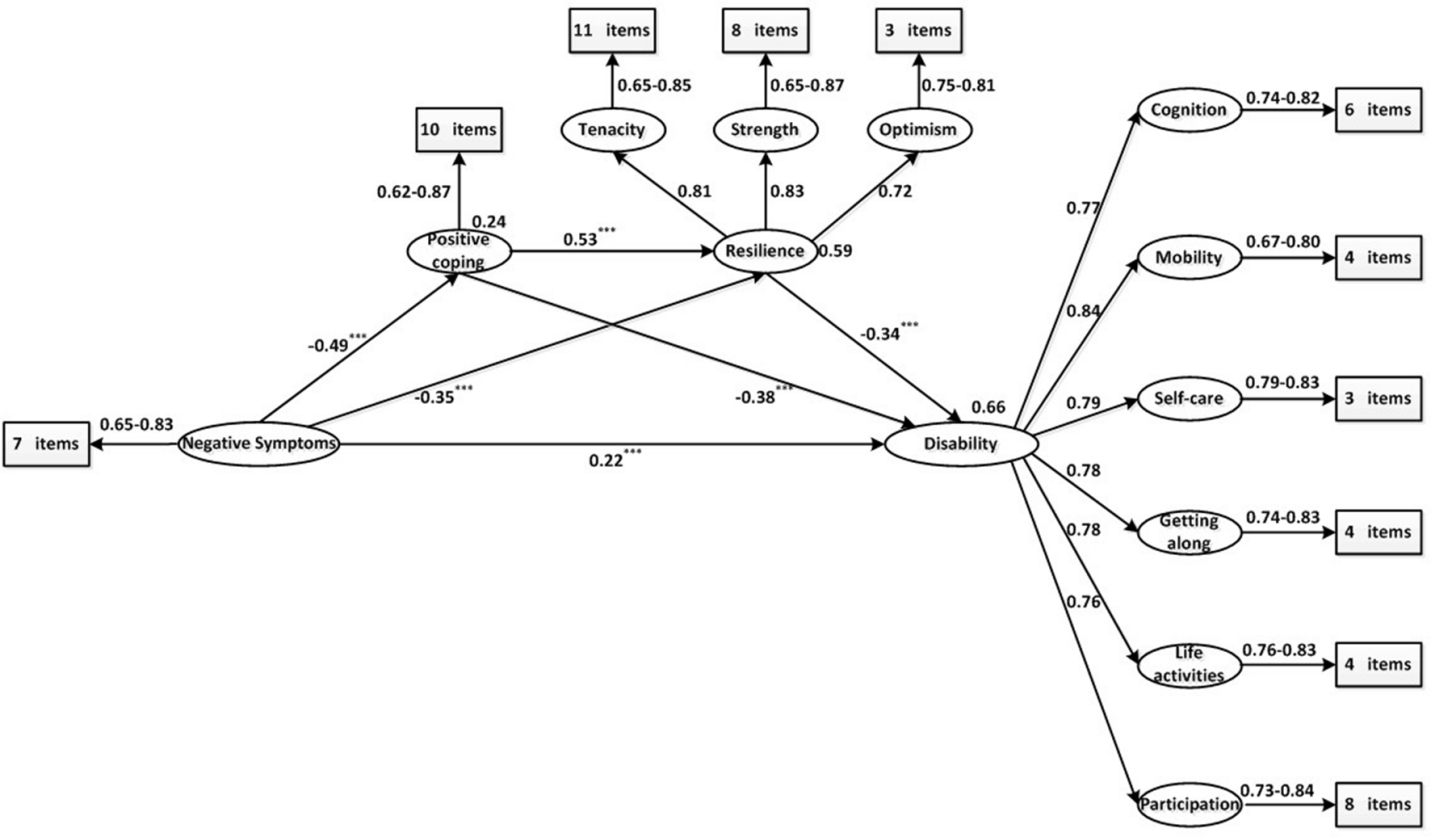
Result of the proposed schizophrenia disability distal mediation model. The ellipses represent latent variables. The rectangles represent observed variables. All path coefficients are standardized. The squared multiple correlation (R^2^) value for the dependent variable appears above the ellipses. *** *p <* 0.001.

Following the measurement model, a structural equation model of disability in schizophrenia was developed to test how well the proposed model fit the collected data. The results of the SEM for our distal mediation model are displayed in **Figure 2**. In the model, the exogenous variable of negative symptoms explained 24% of the variation in positive coping. For resilience, the model explained 59% of the variation in negative symptoms and positive coping. Finally, positive coping and resilience, together with negative symptoms, explained 66% of the variation in disability.

The bootstrapped unstandardized indirect effect was 0.319, with a 95% confidence interval between 0.317 and 0.481, and the direct effect was 0.224, with a 95% confidence interval between 0.112 and 0.330; zero was not included among the lower and upper bounds, indicating that the data are consistent with a partial mediation model (43). None of the asymmetric confidence intervals included a 0 value, indicating that all of the specific indirect effects were established (for details, see **Table 4**). The ratio of the indirect effect to the total effect was 0.63, indicating that positive coping and resilience accounted for approximately two-thirds of the effect of negative symptoms on disability. Among the three specific indirect effects, positive coping was the most vital mediator between negative symptoms and disability based on the ratios of the specific indirect effect to the total indirect effect: the mediation path with only positive coping (48%), the mediation path with only resilience (30%), and the mediation path with both positive coping and resilience (23%). The model was well-fitted to the observed data, with the following goodness-of-fit statistics: χ*^2^* */df* = 1.257, CFI = 0.969, GFI = 0.844, IFI = 0.969, TLT = 0.968, and a 90% confidence interval for the RMSEA = 0.05; 0.06.

**Table 4:** Results of bootstrap and PRODCLIN testing.

Variance	Point estimates	Bootstrapping Bias-corrected 95%CI		Mackinnon PRODCLIN 95%CI		Ratio (%)*
		Lower	Upper	Lower	Upper	
Negative symptoms → Disability	Indirect effect 0.391	0.317	0.481	NS→PC→DI		47.6
				0.079	0.227	
				NS→RE→DI		30.4
				0.04	0.168	
	Direct effect 0.224	0.112	0.33	NS→PC→RE		22.6
				-0.426	-0.187	
				PC→RE→DI		
				-0.194	-0.056	

2,000 bootstrap samples.

NS, negative symptoms; PC, positive coping; RE, resilience; DI, disability; CI, confidence interval.

*The ratio of the specific indirect effect to the total indirect effect.

## Discussion

To the best of our knowledge, our research is the first to establish a mediation model to simultaneously specify the mediating effect of positive coping and resilience on the negative symptoms–disability relationship in patients with schizophrenia. The schizophrenia mediation model explained 66% of the variation in disability.

Our study confirmed that positive coping partly mediated the relationship between negative symptoms and disability in schizophrenia, in line with Meyer (44). We also confirmed that resilience can serve as another important mediator between negative symptoms and disability, which is consistent with the study by Galderisi et al. (6). Resilience reflects the ability to bounce back from stressful or traumatic life experiences rather than adapting passively to the context. The positive predictive role of resilience in functional outcomes and life satisfaction have been clarified in some study populations, such as adolescents, adults (45), and older geriatric patients with multiple comorbidities after orthopedic surgery (46). The identification and development of personal resources, such as resilience and positive coping, help patients with schizophrenia obtain functional recovery, which is difficult to achieve by relying only on pharmacological therapy. However, another study found that resilience was only associated with social functioning and not with the severity of symptoms (7). In this study, psychiatric symptom severity scores were calculated as a composite score based on several items extracted from the positive symptoms, negative symptoms, and general symptoms subscales, which was included in the final SEM as an observed variable. This procedure may have masked specific predictor-mediator-outcome links (47). Another reason for the difference may have been transcultural differences in resilience (48). This possibility sheds light on the need for future studies to develop culture-specific psychosocial intervention programs to prevent schizophrenia-related disability.

The indirect effect of negative symptoms can also be passed on to disability sequentially through positive coping and resilience. These results may be interpreted as indicating that schizophrenia patients with severe negative symptoms are likely to feel unable to manage stress and are therefore more inclined to ignore their problems rather than using positive coping, which depends on attentional volition and adequate cognitive function (49). This deficit makes it more difficult for patients with schizophrenia to adapt to circumstances, to develop and maintain solid interpersonal relationships, or to bounce back from a negative situation. Over time, patients become more isolated and unable to share family responsibilities or live on their own, and disability ultimately develops.

Although both coping and resilience are related to responses to stress, these concepts are distinct. Positive coping involves a set of skills, whereas resilience emphasizes the ability to adapt to and bounce back from extremely unfavorable circumstances. Moreover, the acceptable discriminant validity suggests that positive coping and resilience can be validly measured as different variables that are distinct from negative symptoms and disability, yet related to them. Therefore, positive coping and resilience not only coexist but are also significantly related. Distinguishing between positive coping and resilience may improve our understanding of these mediators, which in turn can enhance our understanding of the pathways of disability in schizophrenia.

Our findings have clinical implications. As any clinician knows, it is very difficult to treat the negative symptoms of antipsychotic medications. Antipsychotic treatment may produce an adverse effect on long-term outcomes by reducing patients’ brain volume and cognitive function (50). According to our results, resilience and positive coping may play protective roles in the process of preventing disability. It may be possible to prevent the damage caused by negative symptoms for patients with schizophrenia by providing targeted interventions to increase positive coping and resilience. In our distal mediation model, positive coping was significantly positively related to resilience, indicating that positive coping may enhance resilience in patients with schizophrenia. Moreover, the ratio of the indirect effect of positive coping to the total indirect effect was 47.6%, higher than that of resilience (30.4%). The aforementioned causes indicate that positive coping should be adequately addressed in integrated interventions for schizophrenia and that treatment should sufficiently utilize the catalytic role of positive coping in resilience. However, this finding does not mean that interventions targeting resilience are not important for preventing disability in patients with schizophrenia. To optimize the protective effect of positive coping on disability in patients with schizophrenia, we also need to promote resilience, which is more proximal to the dependent variable disability. Overall, we suggest the need to integrate positive coping and resilience-targeted intervention into the schizophrenia health management model to sufficiently utilize patients’ personal resources to prevent disability.

This study is not free of limitations. In this study, positive coping, resilience, and disability were assessed based on self-reports from patients with schizophrenia who had insight deficits to different degrees and may have underestimated their level of impairment (51). However, the validity of self-reported outcomes has been reported in some studies (51, 52). Moreover, some researchers have emphasized the importance of considering the self-reported outcomes of patients with schizophrenia in both research and treatment (53, 54). All of the patients had been hospitalized for at least 3 months before their inclusion in our study, and patients who could not complete all of the assessments for any reason were excluded. Therefore, it may not be possible to extrapolate our results to such patients. In addition, a study on resilience in older adults conducted by Callegari et al. (55) found that the level of resilience of older adults who lived in a nursing home, especially older adults suffering from psychiatric disorders, was significantly lower compared with those who lived at home. The results indicated that resilience skills and individual mental resources to face adversities in life may be reduced by institutionalization and psychiatric disease. Institutionalized hospitalization may cause the resilience of patients with psychiatric disorders to be weaker, which is not conducive to recovery. Therefore, to avoid institutionalization, adequate social support may be important when possible. Finally, we would like to acknowledge that the causality of the investigated variables cannot be confirmed because of the cross-sectional design of our study. It seems possible that deficits in positive coping may also have an adverse effect on negative symptoms. Patients with schizophrenia have difficulties using positive coping and usually cannot efficiently handle the stress of daily life, and there is no consensus regarding which coping strategies are most effective (56). Over time, patients become unable to tolerate stressful environments, which leads to an exacerbation of negative symptoms. Thus, we encourage future investigations to clarify the causal relationship between coping and negative symptoms using dynamic long-term studies.

To conclude, we clearly demonstrate that positive coping and resilience are two key causal mediators of the negative symptoms–disability relationship, and we provide new information regarding the complex relationship between negative symptoms and disability in schizophrenia. Furthermore, we found that the indirect effect of positive coping is relatively more important than that of resilience. Future clinical interventions to prevent schizophrenia-related disability can appropriately increase the proportion of interventions that target positive coping to optimize the buffering effect of positive coping and resilience.

## Data Availability

All datasets generated for this study are included in the manuscript.

## Ethics Statement

All the participants received a complete description of our study and provided written informed consent. Our study received approval from the ethics committee of Harbin Medical University (Daqing) and strictly followed the principles of the Declaration of Helsinki.

## Author Contributions

HC, JX, YM, and YZ conceived and designed the experiments. HC, JX, YM, LS, and YS conducted the experiments and collected data. HC and JX analyzed the results. HC wrote the main manuscript text. All of the authors reviewed the manuscript.

## Funding

This research was supported financially by the National Natural Science Foundation of China, NFSC 71673070.

## Conflict of Interest Statement

The authors declare that the research was conducted in the absence of any commercial or financial relationships that could be construed as a potential conflict of interest.

## References

[1] Harvey PD Assessment of everyday functioning in schizophrenia: implications for treatments aimed at negative symptoms. Schizophr Res (2013) 150:353–5. 10.1016/j.schres.2013.04.022 PMC3825780 23668973

[2] Zipursky RB Reilly TJ Murray RM The myth of schizophrenia as a progressive brain disease. Schizophr Bull (2013) 39:1363–72. 10.1093/schbul/sbs135 PMC3796078 23172002

[3] Robertson BR Prestia D Twamley EW Patterson TL Bowie CR Harvey PD Social competence versus negative symptoms as predictors of real world social functioning in schizophrenia. Schizophr Res (2014) 160:136–41. 10.1016/j.schres.2014.10.037 PMC4258126 25468184

[4] Ventura J Subotnik KL Gitlin MJ Gretchen-Doorly D Ered A Villa KF Negative symptoms and functioning during the first year after a recent onset of schizophrenia and 8 years later. Schizophr Res (2015) 161:407–13. 10.1016/j.schres.2014.10.043 PMC4308531 25499044

[5] Wunderink L Sytema S Nienhuis FJ Wiersma D Clinical recovery in first-episode psychosis. Schizophr Bull (2009) 35:362–9. 10.1093/schbul/sbn143 PMC2659307 18990715

[6] Galderisi S Rossi A Rocca P Bertolino A Mucci A Bucci P The influence of illness-related variables, personal resources and context-related factors on real-life functioning of people with schizophrenia. World Psychiatry (2014) 13:275–87. 10.1002/wps.20167 PMC4219069 25273301

[7] Rossi A Galderisi S Rocca P Bertolino A Mucci A Rucci P The relationships of personal resources with symptom severity and psychosocial functioning in persons with schizophrenia: results from the Italian network for research on psychoses study. Eur Arch Psychiatry Clin Neurosci (2017) 267:285–94. 10.1007/s00406-016-0710-9 27381016

[8] Aburn G Gott M Hoare K What is resilience? An integrative review of the empirical literature. J Adv Nurs (2016) 72:980–1000. 10.1111/jan.12888 26748456

[9] Deng M Pan Y Zhou L Chen X Liu C Huang X Resilience and cognitive function in patients with schizophrenia and bipolar disorder, and healthy controls. Front Psychiatry (2018) 9:279. 10.3389/fpsyt.2018.00279 30008678 PMC6033957

[10] Gooding PA Littlewood D Owen R Johnson J Tarrier N Psychological resilience in people experiencing schizophrenia and suicidal thoughts and behaviours. J Ment Health (2017) 28:1–7. 10.1080/09638237.2017.1294742 28635432

[11] Poloni N Zizolfi D Ielmini M Pagani R Caselli I Diurni M A naturalistic study on the relationship among resilient factors, psychiatric symptoms, and psychosocial functioning in a sample of residential patients with psychosis. Psychol Res Behav Manag (2018) 11:123–31. 10.2147/PRBM.S159571 PMC5903837 29695941

[12] Feder A Nestler EJ Charney DS Psychobiology and molecular genetics of resilience. Nat Rev Neurosci (2009) 10:446–57. 10.1038/nrn2649 PMC2833107 19455174

[13] Harrow M Jobe TH Factors involved in outcome and recovery in schizophrenia patients not on antipsychotic medications: a 15-year multifollow-up study. J Nerv Ment Dis (2007) 195:406–14. 10.1097/01.nmd.0000253783.32338.6e 17502806

[14] Torgalsbøen AK Rund BR Maintenance of recovery from schizophrenia at 20-year follow-up: what happened? Psychiatry (2010) 73:70–83. 10.1521/psyc.2010.73.1.70 20235619

[15] Zizolfi D Poloni N Caselli I Ielmini M Lucca G Diurni M Resilience and recovery style: a retrospective study on associations among personal resources, symptoms, neurocognition, quality of life and psychosocial functioning in psychotic patients. Psychol Res Behav Manag (2019) 12:385–95. 10.2147/PRBM.S205424 PMC6549482 31213935

[16] Mihali A Subramani S Kaunitz G Rayport S Gaisler-Salomon I Modeling resilience to schizophrenia in genetically modified mice: a novel approach to drug discovery. Expert Rev Neurother (2012) 12:785–99. 10.1586/ern.12.60 PMC3509194 22853787

[17] Taylor SE Stanton AL Coping resources, coping processes, and mental health. Annu Rev Clin Psychol (2007) 3:377–401. 10.1146/annurev.clinpsy.3.022806.091520 17716061

[18] Holubova M Prasko J Latalova K Ociskova M Grambal A Kamaradova D Are self-stigma, quality of life, and clinical data interrelated in schizophrenia spectrum patients? a cross-sectional outpatient study. Patient Prefer Adherence (2016) 10:265–74. 10.2147/PPA.S96201 PMC4786060 27019596

[19] Boschi S Adams RE Bromet EJ Lavelle JE Everett E Galambos N Coping with psychotic symptoms in the early phases of schizophrenia. Am J Orthopsychiatry (2000) 70:242–52. 10.1037/h0087710 10826036

[20] Gloria CT Steinhardt MA Relationships among positive emotions, coping, resilience and mental health. Stress Health (2016) 32:145–56. 10.1002/smi.2589 24962138

[21] Folkman S Lazarus RS Manual for the Ways of Coping Questionnaire. Palo Alto, CA: Consulting Psychologists Press (1988). 10.1037/t06501-000

[22] Carver CS Scheier MF Weintraub JK Assessing coping strategies: a theoretically based approach. J Pers Soc Psychol (1989) 56:267–83. 10.1037/0022-3514.56.2.267 2926629

[23] Xie Y Reliability and validity of the simplified coping style questionnaire. Chin J Clin Psychol (1998) 6:114–5.

[24] Yu Y Peng L Chen L Long L He W Li M Resilience and social support promote posttraumatic growth of women with infertility: the mediating role of positive coping. Psychiatry Res (2014) 215:401–5. 10.1016/j.psychres.2013.10.032 24368061

[25] Connor KM Davidson JR Development of a new resilience scale: the Connor-Davidson resilience scale (CD-RISC). Depress Anxiety (2003) 18:76–82. 10.1002/da.10113 12964174

[26] Yu X Zhang J Factor analysis and psychometric evaluation of the Connor-Davidson resilience scale (CD-RISC) with Chinese people. Soc Behav Pers (2007) 35:19–30. 10.2224/sbp.2007.35.1.19

[27] World Health Organization World Health Organization Disability Assessment Schedule: WHODAS II. Phase 2 Field Trials. Health Services Research. Geneva, Switzerland: WHO (2000).

[28] Peuskens J Gorwood P How are we assessing functioning in schizophrenia? A need for a consensus approach. Eur Psychiatry (2012) 27:391–5. 10.1016/j.eurpsy.2011.02.013 21632218

[29] Ustun TB Kostanjesek N Chatterji S Rehm J Measuring Health and Disability: Manual for WHO Disability Assessment Schedule (WHODAS 2.0). Geneva, Switzerland: WHO (2010).

[30] Chiu TY Yen CF Chou CH Lin JD Hwang AW Liao HF Development of traditional Chinese version of world health organization disability assessment schedule 2.0 36 – item (WHODAS 2.0) in Taiwan: validity and reliability analyses. Res Dev Disabil (2014) 35:2812–20. 10.1016/j.ridd.2014.07.009 25094056

[31] Cudeck R Analysis of correlation matrices using covariance structure models. Psychol Bull (1989) 105:317–27. 10.1037/0033-2909.105.2.317

[32] Anderson JC Gerbing DW Structural equation modeling in practice: a review of the two-step approach. Psychol Bull (1988) 103:411–23. 10.1037//0033-2909.103.3.411

[33] Schumacker RE Lomax RG A Beginner’s Guide to Structural Equation Modeling. New York, NY: Routledge (1996).

[34] Hu LT Bentler PM Cutoff criteria for fit indexes in covariance structure analysis: conventional criteria versus new alternatives. Struct Equ Modeling (1999) 6:1–55. 10.1080/10705519909540118

[35] Kline RB Principles and Practice of Structural Equation Modeling. New York, NY: Guilford Publications (2015).

[36] Holbert RL Stephenson MT The importance of indirect effects in media effects research: testing for mediation in structural equation modeling. J Broadcast Electron Media (2003) 47:556–72. 10.1207/s15506878jobem4704_5

[37] Mackinnon DP Fritz MS Williams J Lockwood CM Distribution of the product confidence limits for the indirect effect: program PRODCLIN. Behav Res Methods (2007) 39:384–9. 10.3758/BF03193007 PMC2819369 17958149

[38] Fritz MS Mackinnon DP Required sample size to detect the mediated effect. Psychol Sci (2007) 18:233–9. 10.1111/j.1467-9280.2007.01882.x PMC2843527 17444920

[39] Hair JF Black WC Babin BJ Multivariate Data Analysis: A Global Perspective. Upper Saddle River: Prentice Hall (2010).

[40] Hooper D Coughlan J Mullen M Structural equation modelling: guidelines for determining model fit. J Res Natl Inst Stand Technol (2008) 6:53–60. 10.0000/PMID35188134

[41] Fornell C Larcker DF Evaluating structural equation models with unobservable variables and measurement error. J Mark Res (1981) 18:39–50. 10.1177/002224378101800104

[42] Marsh HW The hierarchical structure of self-concept and the application of hierarchical confirmatory factor analysis. J Educ Meas (1987) 24:17–39. 10.1111/j.1745-3984.1987.tb00259.x

[43] Frazier PA Tix AP Barron KE Testing moderator and mediator effects in counseling psychology research. J Mark Res (2004) 51:115–34. 10.1037/0022-0167.51.1.115

[44] Meyer B Coping with severe mental illness: relations of the brief COPE with symptoms, functioning, and well-being. J Psychopathol Behav Assess (2001) 23:265–77. 10.1023/A:1012731520781

[45] Callegari C Bertu L Lucano M Ielmini M Braggio E Vender S Reliability and validity of the Italian version of the 14-item Resilience Scale. Psychol Res Behav Manag (2016) 9:277–84. 10.2147/PRBM.S115657 PMC5055039 27757055

[46] Rebagliati GA Sciume L Iannello P Mottini A Antonietti A Caserta VA Frailty and resilience in an older population. The role of resilience during rehabilitation after orthopedic surgery in geriatric patients with multiple comorbidities. Funct Neurol (2016) 31:171–7. 10.11138/FNeur/2016.31.3.171 PMC5115232 27678211

[47] Schmidt SJ Mueller DR Roder V Social cognition as a mediator variable between neurocognition and functional outcome in schizophrenia: empirical review and new results by structural equation modeling. Schizophr Bull (2011) 37 Suppl 2:S41–54. 10.1093/schbul/sbr079 PMC3160114 21860046

[48] Hofer A Mizuno Y Frajo-Apor B Kemmler G Suzuki T Pardeller S Resilience, internalized stigma, self-esteem, and hopelessness among people with schizophrenia: cultural comparison in Austria and Japan. Schizophr Res (2016) 171:86–91. 10.1016/j.schres.2016.01.027 26805413

[49] Mingrone C Montemagni C Sandei L Bava I Mancini I Cardillo S Coping strategies in schizoaffective disorder and schizophrenia: differences and similarities. Psychiatry Res (2016) 244:317–23. 10.1016/j.psychres.2016.06.059 27517342

[50] MacKenzie NE Kowalchuk C Agarwal SM Costa-Dookhan KA Caravaggio F Gerretsen P Antipsychotics, metabolic adverse effects, and cognitive function in schizophrenia. Front Psychiatry (2018) 9:622. 10.3389/fpsyt.2018.00622 30568606 PMC6290646

[51] Bell M Fiszdon J Richardson R Lysaker P Bryson G Are self-reports valid for schizophrenia patients with poor insight? relationship of unawareness of illness to psychological self-report instruments. Psychiatry Res (2007) 151:37–46. 10.1016/j.psychres.2006.04.012 17343920

[52] Palmer BW Martin AS Depp CA Glorioso DK Jeste DV Wellness within illness: happiness in schizophrenia. Schizophr Res (2014) 159:151–6. 10.1016/j.schres.2014.07.027 PMC4928639 25153363

[53] Ethan B The missing voice of patients in drug-safety reporting. NEJM (2010) 362:865–9. 10.1056/NEJMp0911494 PMC3031980 20220181

[54] Riley WT Paul P David C Application of the national institutes of health patient-reported outcome measurement information system (PROMIS) to mental health research. J Ment Health Policy Econ (2011) 14:201–8. 10.1002/chin.201152096 PMC3705221 22345362

[55] Callegari C Bertù L Caselli I Isella C Ielmini M Bonalumi C Resilience in older adults: influence of the admission in nursing home and psychopathology. Neuropsychiatry J (2016) 6:117–23. 10.4172/Neuropsychiatry.1000129

[56] Austenfeld JL Stanton AL Coping through emotional approach: a new look at emotion, coping, and health-related outcomes. J Pers (2004) 72:1335–63. 10.1111/j.1467-6494.2004.00299.x 15509285

